# Polymorphic Ventricular Tachycardia (PMVT) in a Patient With Troponin-Negative Chest Pain: A Case Report and Literature Review

**DOI:** 10.7759/cureus.85016

**Published:** 2025-05-29

**Authors:** Abhimanyu Baruah Baruah, Weje Chituru, Chin Soo, Syed Mustafa, Adnan Ahmed, Jhiamluka Solano

**Affiliations:** 1 Integrative Medicine, Northern Lincolnshire and Goole NHS Foundation Trust, Scunthorpe, GBR; 2 Cardiology, Northern Lincolnshire and Goole NHS Foundation Trust, Grimsby, GBR; 3 Cardiology, Hull University Hospital, Castle Hill Hospital, Hull, GBR; 4 Resident Doctor Committee, Royal College of Physicians, London, GBR; 5 Education Committee, Academy of Medical Educators, Cardiff, GBR; 6 Cardiology, Scunthorpe General Hospital, Scunthorpe, GBR

**Keywords:** acute coronary syndromes, cardiac arrythmia, chronic coronary syndrome (ccs), left anterior descending artery, ventricular tachycardia (vt)

## Abstract

Polymorphic ventricular tachycardia (PMVT) is a potentially life-threatening arrhythmia, typically associated with acute myocardial ischemia or inherited channelopathies. We present a case of PMVT in the context of critical coronary artery disease (CAD) but without biomarker-evident myocardial injury, such as troponin elevation. We present a case of a 61-year-old man with critical left anterior descending (LAD) artery stenosis who developed symptomatic PMVT. This case highlights the paradox of negative troponin values despite severe coronary pathology and a life-threatening arrhythmia. Despite initial normal troponin levels, the patient’s recurrent chest pain and arrhythmias were ultimately attributed to critical LAD stenosis, which was successfully treated with percutaneous coronary intervention (PCI). This case emphasizes the importance of considering high-risk coronary disease, particularly in patients with exertional symptoms and arrhythmias, even when initial biomarkers may be reassuring. It underscores the importance of considering ischemia even when troponin levels are normal, particularly in patients with unstable angina. The case illustrates the limitations of relying solely on troponin for risk stratification in acute coronary presentations and supports the need for comprehensive clinical and electrocardiographic evaluation.

## Introduction

Chest pain is a common presenting complaint in emergency departments, necessitating rapid risk stratification to identify patients at risk of acute coronary syndromes (ACS) [1-4]. While cardiac troponins are central to the diagnostic algorithm, they are less able to detect severe underlying coronary pathology without myocardial injuries (such as unstable angina, which requires a high index of clinical suspicion), and old myocardial scars or arrhythmogenic cardiomyopathy, which predispose to life-threatening ventricular arrhythmias.

Critical ostial stenoses of the left anterior descending (LAD) artery, often referred to as the “widow-maker,” are of particular clinical concern due to the large myocardial territory at risk of ischemic events. Undetected ostial LAD stenoses often serve as a substrate for malignant ventricular arrhythmias, sudden cardiac deaths, or significant myocardial injury despite preserved left ventricular function [5]. Polymorphic ventricular tachycardia (VT) (PMVT) in a structurally normal heart should prompt a thorough search for reversible ischemic triggers, especially in the presence of exertional symptoms or syncope [6]. However, its occurrence in the absence of myocardial injury, as evidenced by normal cardiac biomarkers, is rare and underreported.

We report the case of a 61-year-old man with preserved bi-ventricular function and multiple prior hospital presentations for chest pain, who was ultimately found to have a critical ostial LAD stenosis responsible for symptomatic PMVT. This case highlights the importance of comprehensive evaluation in patients with recurrent symptoms and negative initial investigations, and it illustrates the limitations of relying solely on troponin kinetics in identifying life-threatening coronary artery disease (CAD).

## Case presentation

A 61-year-old man with a medical history of hypertension, hypercholesterolemia, and severe scoliosis presented to the emergency department with exertional chest pain. It was described as a pressure-like sensation encircling his chest, radiating to the neck and left arm. The episode lasted approximately 30 minutes and was accompanied by sweating, palpitations, and moderate dyspnea.

He reported a history of intermittent chest pain over the past decade. A year prior, a treadmill exercise stress test (up to 8.62 METs (metabolic equivalents), being able to do up to nine minutes and three stages of the Bruce protocol with good hemodynamic response and no chest pain/ST-T segment changes) was negative. However, the frequency of exertional chest pain had increased over the past two years. He recalled a previous episode of exertional syncope five years prior, which was preceded by the sudden onset of dyspnea, but he did not seek medical attention. He had a family history of non-premature ischemic heart disease and had recently ceased smoking.

**Figure 1:**
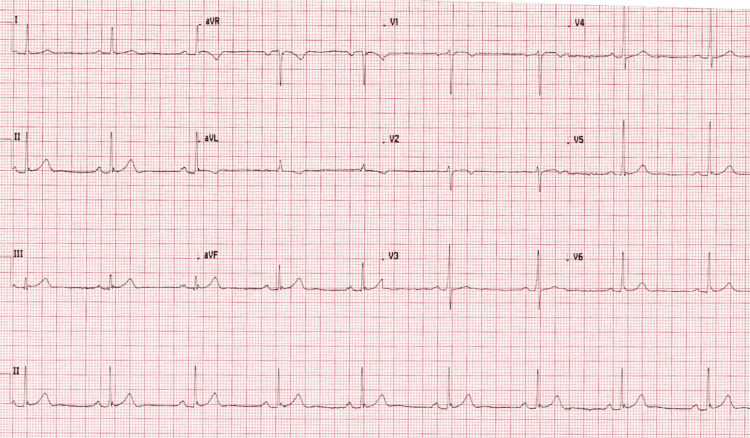
Twelve-lead ECG showing sinus bradycardia. Sinus bradycardia is defined on an electrocardiogram (ECG) as a sinus rhythm with a heart rate of less than 60 beats per minute (bpm) in adults.

On initial assessment, vital signs were as follows: heart rate 56 beats per minute (bpm), blood pressure 116/76 mmHg, respiratory rate 16 breaths per minute, and oxygen saturation 98% on room air. A 12-lead electrocardiogram (ECG) demonstrated sinus bradycardia at 54 bpm with a QTc of 373 ms without any ST-segment or repolarization abnormalities (see Figure 1). Initial high-sensitivity troponin was 12 ng/L, with a repeat value of 11 ng/L at four hours. Apart from raised serum low-density lipoprotein (LDL), full blood count, renal function, and electrolytes were normal (see Table 1).

**Table 1:** Initial blood tests on admission. Hb: hemoglobin; eGFR: estimated glomerular filtration rate; LDL: low-density lipoprotein

Investigation	Values	Normal ranges
Hb	149 g/L	132-170 g/L
eGFR	79 mL/min	90-200 mL/min
HbA1c	3.3%	<5.7%
LDL	4.1 mmol/L	<3.0 mmol/L
K^+^	4.6 mmol/L	3.5-5.3 mmol/L
Mg	0.89 mmol/L	0.70-1.0 mmol/L
Ca	2.54 mmol/L	2.2-2.6 mmol/L

**Figure 2:**
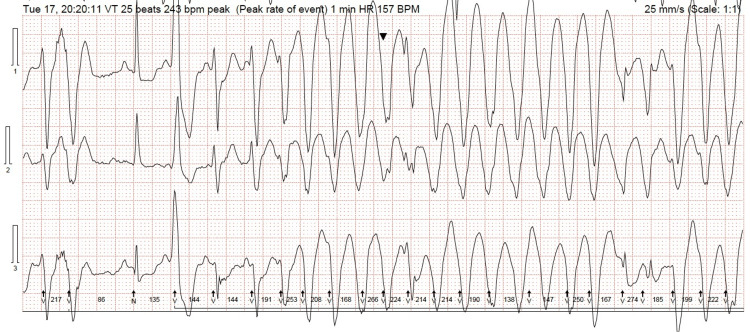
The Holter recording showing sinus rhythm with multiple episodes of polymorphic wide complex tachycardia consistent with ventricular tachycardia. The arrows (bottom) and arrowhead (top) that point at the QRS complex represent R-wave detections by the Holter software. The top arrowhead represents the patient-triggered event marker.

A few months prior to the admission, the patient was referred for Holter ECG monitoring due to an episode of collapse associated with dyspnea and dizziness. The Holter recording revealed a sinus rhythm with multiple episodes of polymorphic wide-complex tachycardia, consistent with VT, with a maximum duration of 33 beats and a maximum rate of 243 bpm. There were multifocal ventricular ectopics, including 11 episodes of ventricular salvos, the longest of which comprised 4 beats at a maximum rate of 207 bpm, as well as 29 triplets and 70 couplets. Additionally, there were two episodes of trigeminy, with the longest lasting four cycles, and 24 episodes of bigeminy, with the longest persisting for 16 cycles (see Figure 2). These arrhythmias correlated with self-reported symptoms of chest and head pressure, dizziness, palpitations, and paresthesia affecting the limbs. The Holter monitor revealed a high burden of polymorphic ventricular ectopy, consistent with increased arrhythmic risk. Additionally, the patient had recurrent hospital admissions with chest pain on three different occasions over three months.

During the admission, transthoracic echocardiography demonstrated normal bi-ventricular sizes and systolic function with left ventricular ejection fraction (LVEF) greater than 55% as assessed by single-plane Simpson’s rule. There was mild concentric left ventricular hypertrophy (maximum wall thickness of 14 mm) secondary to hypertension (see Video 1). Inpatient computer tomography of the carotid and intracranial angiography excluded dissection or significant stenosis.

**Video 1:** Transthoracic echocardiogram (TTE). Apical four-chamber view with dedicated left ventricular views to highlight the described left ventricular hypertrophy (LVH). LV: left ventricle; RV: right ventricle; LA: left atrium; RA: right atrium

During admission, an uncomplicated invasive coronary angiography via the right radial artery using standard catheters revealed severe 99% ostial stenosis of the LAD artery, extending 48 mm in length, with pre-procedure thrombolysis in myocardial infarction (MI) (TIMI) III flow and good distal run-off; fractional flow reserve (FFR) was not done due to the severity of the lesion (see Video 2). The LAD lesion was considered the likely arrhythmic substrate following discussion with electrophysiologists and interventionists. The lesion was successfully treated with percutaneous coronary intervention (PCI) with balloon angioplasty and placement of a drug-eluting stent (3.5 x 48, XIENCE), and FFR of the right coronary artery was done due to spasms noted, which was normal (0.95). No intravascular ultrasound (IVUS)/optical coherence tomography (OCT) imaging was done. Dual antiplatelet therapy was commenced with ticagrelor 90 mg BD for one year and lifelong aspirin 75 mg, along with atorvastatin 40 mg and bisoprolol 1.25 mg. Repeat Holter ECG monitoring was scheduled with an outpatient cardiology clinic review at three months to assess any further PMVT burden. 

**Video 2:** Percutaneous coronary intervention (PCI) to the left anterior descending (LAD) coronary artery (A) Left anterior oblique (LAO) view showing severe 99% ostial stenosis of the LAD artery, extending 48 mm in length, with a pre-procedure TIMI III flow and good distal run-off (arrow); (B) LAO view showing guidewire through the lesion; (C) LAO close view of the balloon and stent deployment; (D) right anterior oblique (RAO) view showing post-PCI result. TIMI: thrombolysis in myocardial infarction

## Discussion

Troponin is a key biomarker of myocardial injury used to diagnose acute MI. However, it shows a significant false negative rate in its correlation with underlying CAD. In one study, 61% (n = 542/895) of patients who underwent angiography had elevated troponin values, of whom 94% had evidence of significant angiographic CAD. Of the remaining 353 patients with negative troponin, 79% had significant CAD [7]. A positive troponin without evidence of CAD may be secondary to myocardial injury (type II MI) or coronary microvascular disease with normal coronary angiography.

Our case presents a situation where high-sensitivity troponin testing was negative in the presence of significant ischemia. Notably, several factors, including analytical interference, are known to lead to false negatives and positives (see Table 2) [8]. To note that in our case, no clinical or laboratory evidence suggested the presence of analytical interferences such as heterophile antibodies or sample hemolysis in this patient, supporting the validity of the troponin results reported. While such interferences are important considerations in cases of discordant troponin results, their absence in this scenario underscores the diagnostic challenge posed by ischemia without myocyte necrosis.

**Table 2:** Possible causes for false-negative and false-positive troponin results.

False-negative troponin	False-positive troponin
Hyperbilirubinemia	Heterophile antibodies
Lipemia	Myopathies
Biotin supplementation (vitamin B7)	Rheumatoid factor
Cardiac troponin antibodies	Fibrin interference
Hemolysis	Hemolysis
Analyzer malfunction	Elevated alkaline phosphatase
	Analyzer malfunction

Another possible cause of false negatives is early sampling. The release of cardiac troponin follows a time-dependent curve [9]. In a paper published in the European Heart Journal [10], when using sex-specific 99th centile (16 ng/L in women; 34 ng/L in men) thresholds, the sensitivity of troponin-T to accurately rule out MI appears to be especially low in early presenters (<3 hours), at 71.4%. It concluded that the 99th centile should not be used to rule out MI. While our patient did not have a MI, the conclusion remains that troponin-T testing with the 99th percentile may not be adequate in ruling out significant ischemia. Krüger et al. studied the release kinetics of various biomarkers in short-lasting myocardial ischemia. They found that serum troponin-T levels remained unchanged from baseline in severe myocardial ischemia with a duration of up to 5.8 ± 1.6 minutes [11]. In this case, serial troponin measurements were timed shortly after symptom onset and during periods of transient ischemia, which likely explains the absence of detectable biomarker elevation despite significant CAD and documented arrhythmia. This highlights the importance of correlating troponin dynamics with clinical presentation and ischemic duration when interpreting biomarker results, particularly in unstable angina or ischemia without infarction.

A comprehensive approach is necessary for assessing chest pain or PMVT despite negative serial troponin-T tests. Co-morbidities (such as rheumatoid arthritis) and traditional risk factors that predispose to CAD must be carefully considered. Factors such as diabetes and age are the strongest predictors of CAD [12]. ECG changes, such as Q-waves in the inferior leads and symmetrical T-wave inversions, are independent, highly specific predictors of angiographically confirmed CAD [13]; however, ECG alone as a predictor of CAD shows poor sensitivity [14]. This highlights the need for a holistic and comprehensive approach, which shall include serial ECGs and transthoracic echocardiograms. A diagnosis of unstable angina ought to be considered in one with convincing symptoms and strong cardiac risk factors.

PMVT caused by acute myocardial ischemia is a potentially lethal electrical instability that originates from ischemic regions of the myocardium. Ischemia causes metabolic and ionic derangements in the myocardium, leading to electrical instability via several mechanisms, including slowed conduction velocity, heterogeneous action potential shortening, and dispersion of repolarization across affected myocardial regions [15]. These changes facilitate the development of functional re-entrant circuits and phase 2 re-entry phenomena, creating a substrate that is highly susceptible to the initiation of PMVT. Moreover, ischemia promotes intracellular calcium overload due to impaired calcium handling, leading to delayed afterdepolarizations and triggered activity, particularly at the border zones of ischemic tissue. These arrhythmogenic mechanisms are often exacerbated by autonomic nervous system activation, serum electrolyte disturbances, and residual structural myocardial abnormalities, increasing the risks for recurrent PMVT and sudden cardiac death if re-vascularization or targeted anti-ischemic therapy is not promptly achieved. The PMVT observed in this case likely reflects an ischemic arrhythmic substrate created by the critical LAD stenosis, emphasizing the role of ischemia-induced electrical instability. Understanding the underlying mechanisms informed the decision to proceed with PCI, which aimed to eliminate the arrhythmogenic focus and reduce the risk of recurrent ventricular arrhythmias.

In contrast, non-ischemic causes of PMVT involve distinct molecular and cellular pathophysiological processes. Inherited syndromes such as catecholaminergic PMVT (CPVT) are characterized by mutations primarily in the ryanodine receptor (RyR2) or calsequestrin-2 (CASQ2) genes, resulting in dysfunctional calcium handling under adrenergic stimulation and promoting delayed afterdepolarizations [16]. Similarly, congenital and acquired long QT syndromes predispose to early afterdepolarizations by prolonging ventricular repolarization, often culminating in torsades de pointes. Drug-induced PMVT shares this pathophysiological basis, where pharmacological inhibition of repolarizing potassium currents, particularly I_Kr, extends action potential duration and provokes early afterdepolarizations [17]. Although non-ischemic mechanisms are increasingly recognized, ischemia remains the predominant and most acutely life-threatening cause of PMVT, underscoring the necessity for the rapid identification and management of ischemic substrates in patients presenting with this arrhythmia.

A similar case study was reported in 2010 of a 40-year-old man who had recurrent syncope from PMVT caused by critical obstruction of the RCA. There was no evidence of myocardial injury, ventricular impairment, or electrical abnormalities on ECGs. No further episodes of VT were recorded on a Holter after successful PCI to the right coronary artery [18]. In a Japanese prospective study, PMVT, preceded by transient ST elevation, was identified on Holter monitors in more than 13% of patients with known vasospastic angina. Those with prior PMVT had significantly higher risks of death compared to those without [18]. Another case study described self-terminating PMVT, also preceded by progressive ST-segment elevation, in a man with coronary vasospasms [19]. These case reports, along with the study by Nishizaki et al. [18], underscore the importance of considering CAD even in the absence of typical markers or clear ECG findings. Moreover, unlike some cases with structural heart disease or infarction, this patient’s preserved myocardial integrity highlights the spectrum of clinical presentations and the importance of individualized diagnostic strategies.

Given the increased mortality risks of PMVT due to myocardial ischemia, irrespective of QTc intervals, there shall be a high index of clinical suspicion of undiagnosed CAD, especially in those with exertional chest pain or cardiovascular risk factors [20]. Patients with increasing or worsening episodes of cardiac-sounding chest pain, even in the presence of normal serial serum troponins, shall be treated as acute coronary syndrome (unstable angina) and have coronary arteries evaluated urgently with either invasive or CT coronary angiography to guide further treatments. In patients with stable, less acute symptoms, cardiac magnetic resonance imaging (MRI) (CMR) with a stress perfusion protocol provides a comprehensive assessment of myocardial structure and function, identifies possible fibrosis or scarring, and detects inducible ischemia, aiding prognostication. The absence of inducible ischemia or fibrosis or scar on CMR confers significantly lower risks of cardiovascular deaths or non-fatal MI [2]. While CMR is valuable for assessing myocardial viability and fibrosis in stable patients, its role in this case was limited given the acute presentation and definitive findings on invasive angiography.

Despite the acute resolution of ischemia post-PCI, long-term arrhythmic risk remains a concern, warranting ongoing surveillance through repeat Holter monitoring and clinical follow-up. Consideration of implantable cardioverter defibrillator (ICD) therapy should be individualized based on persistent arrhythmic burden, structural findings, and risk stratification. However, its indication in ischemia-induced PMVT without infarction remains a matter of controversy. In our case, the patient did not meet a Class 1 indication for ICD, as per the 2023 European Society of Cardiology (ESC) guidance for ventricular arrhythmias [21].

## Conclusions

This case demonstrates that critical CAD, such as ostial LAD stenosis, can lead to PMVT and other malignant arrhythmias, even in the absence of troponin elevation. This highlights the potential for troponin alone to delay diagnosis in high-risk patients, particularly when symptoms and non-invasive testing are inconclusive. Despite a falsely negative stress test, the patient’s recurrent presentations with exertional chest pain and syncope, both typical of ischemia, warranted further investigation. Prior Holter monitoring revealed a high burden of ventricular ectopy, which, together with persistent symptoms, supported the decision to pursue invasive angiography. This highlights the importance of a multi-faceted diagnostic approach, guided by clinical judgment, serial ECGs, ambulatory rhythm monitoring, and an appropriate threshold for invasive assessment. Early identification and intervention, such as PCI, are crucial to prevent sudden cardiac death in anatomically high-risk lesions. Post-revascularization, long-term management should include arrhythmia surveillance and risk stratification, with consideration of an ICD if indicated.
